# T lymphocyte engineering with cytokine nanogels for enhanced cancer immunotherapy

**DOI:** 10.1186/2051-1426-3-S2-P54

**Published:** 2015-11-04

**Authors:** Li Tang, Yiran Zheng, Llian Mabardi, Darrell J Irvine

**Affiliations:** 1 Massachusetts Institute of Technology, Cambridge, MA, USA

## Background

Adoptive cell transfer (ACT) with autologous tumor-reactive T cells is a promising strategy in cancer immunotherapy, but treatment of solid tumors is limited by the rapid decline in function of the transplanted T cells. In order to maintain high numbers of viable antigen-specific cytotoxic T cells in tumors, co-administration of supporting immunostimulant agents together with transferred cells is often necessary in clinical practice. However, the high systemic doses of such agents needed to enhance T cell functionality can also result in serious side effects.

## Methods

Here, we developed a carrier-free strategy to deliver cytokines specifically to adoptively transferred T cells for cancer immunotherapy. IL-2-Fc or an IL-15 superagonist were chemically cross-linked with a disulfide linker to form protein nanogels (NGs), which were conjugated to the plasma membrane of ACT T cells.

## Results

These NGs had exceptionally high loading of cytokines (~70 wt%) and released native protein in physiological conditions in a sustained manner through breakdown of the degradable disulfide linker in response to the activated T cell surface reduction activity. Cytokine-NGs were chemically conjugated onto the plasma membrane of donor T cells, enabling continuous pseudo-autocrine release of cytokine for stimulation of transferred CD8+ T cells. Transferred pmel-1 CD8+ T cells with optimized number of NGs conjugated per cell showed enhanced expansion and long persistence in B16F10 tumor bearing mice. Quantification of transferred Thy1.1+CD8+ T cells in tumors at Day 13 showed that T cells with conjugated cytokine-NGs expanded ~80 fold more than the T cells with systemically administered free cytokine.

## Conclusions

We demonstrated that the cytokine-NG-T cell conjugation strategy could augment transferred T cell expansion efficiently and specifically *in vivo*, and thus improve the therapeutic efficacy. This T cell-NG “back pack” approach provides a readily generalizable strategy to provide autocrine protein drug support to donor cells to enhance the safety and efficacy of ACT.

